# The Mobius AIRO mobile CT for image‐guided proton therapy: Characterization & commissioning

**DOI:** 10.1002/acm2.12084

**Published:** 2017-04-24

**Authors:** Jasmine A. Oliver, Omar A. Zeidan, Sanford L. Meeks, Amish P. Shah, Jason Pukala, Patrick Kelly, Naren R. Ramakrishna, Twyla R. Willoughby

**Affiliations:** ^1^ Department of Radiation Oncology UF Health Cancer Center – Orlando Health Orlando FL USA

**Keywords:** AIRO, IGPT, mobile CT, proton therapy

## Abstract

**Purpose:**

The purpose of this study was to characterize the Mobius AIRO Mobile CT System for localization and image‐guided proton therapy. This is the first known application of the AIRO for proton therapy.

**Methods:**

Five CT images of a Catphan^®^504 phantom were acquired on the AIRO Mobile CT System, Varian EDGE radiosurgery system cone beam CT (CBCT), Philips Brilliance Big Bore 16 slice CT simulator, and Siemens SOMATOM Definition AS 20 slice CT simulator. DoseLAB software v.6.6 was utilized for image quality analysis. Modulation transfer function, scaling discrepancy, geometric distortion, spatial resolution, overall uniformity, minimum uniformity, contrast, high CNR, and maximum HU deviation were acquired. Low CNR was acquired manually using the CTP515 module. Localization accuracy and CT Dose Index were measured and compared to reported values on each imaging device. For treatment delivery systems (Edge and Mevion), the localization accuracy of the 3D imaging systems were compared to 2D imaging systems on each system.

**Results:**

The AIRO spatial resolution was 0.21 lp mm^−1^ compared with 0.40 lp mm^−1^ for the Philips CT Simulator, 0.37 lp mm^−1^ for the Edge CBCT, and 0.35 lp mm^−1^ for the Siemens CT Simulator. AIRO/Siemens and AIRO/Philips differences exceeded 100% for scaling discrepancy (191.2% and 145.8%). The AIRO exhibited higher dose (>27 mGy) than the Philips CT Simulator. Localization accuracy (based on the MIMI phantom) was 0.6° and 0.5 mm. Localization accuracy (based on Stereophan) demonstrated maximum AIRO‐kV/kV shift differences of 0.1 mm in the x‐direction, 0.1 mm in the y‐direction, and 0.2 mm in the z‐direction.

**Conclusions:**

The localization accuracy of AIRO was determined to be within 0.6° and 0.5 mm despite its slightly lower image quality overall compared to other CT imaging systems at our institution. Based on our study, the Mobile AIRO CT system can be utilized accurately and reliably for image‐guided proton therapy.

## 1 Introduction

Advances in in‐room computed tomography (CT) scanners and cone‐beam technology have led to the proliferation of CT localization for image guided radiation therapy (IGRT).^1^, ^2^, ^3^, ^4^, ^5^, ^6^, ^7^ These imagers provide improved treatment accuracy over conventional orthogonal imaging allowing for increased precision in radiation delivery. Patient localization accuracy is particularly important in proton beam radiation therapy due to the sharp dose fall‐off compared to conventional x‐ray therapy. Unfortunately, due to the size and geometry of proton therapy units, imaging has largely been limited to orthogonal kV/kV x‐ray systems.^8^ Recently, people have reported on the use of in‐room CT scanners for proton beam radiation therapy localization.^9^, ^10^, ^11^ Some proton vendors are developing technologies for CBCT (Proteus^®^ONE, IBA, Belgium, HITACHI, Tokyo, Japan). To date, stand‐alone in‐room CT scanners utilized have had large footprints and are either not amenable to or cumbersome to use in the more compact proton therapy centers, such as the S250 (Mevion Medical Systems, Littleton, MA, USA).^12^ For these reasons, a small‐footprint mobile CT scanner, commonly used for image‐guided surgery, could be of great value for 3D image‐guided proton therapy (IGPT).

**Figure 1:**
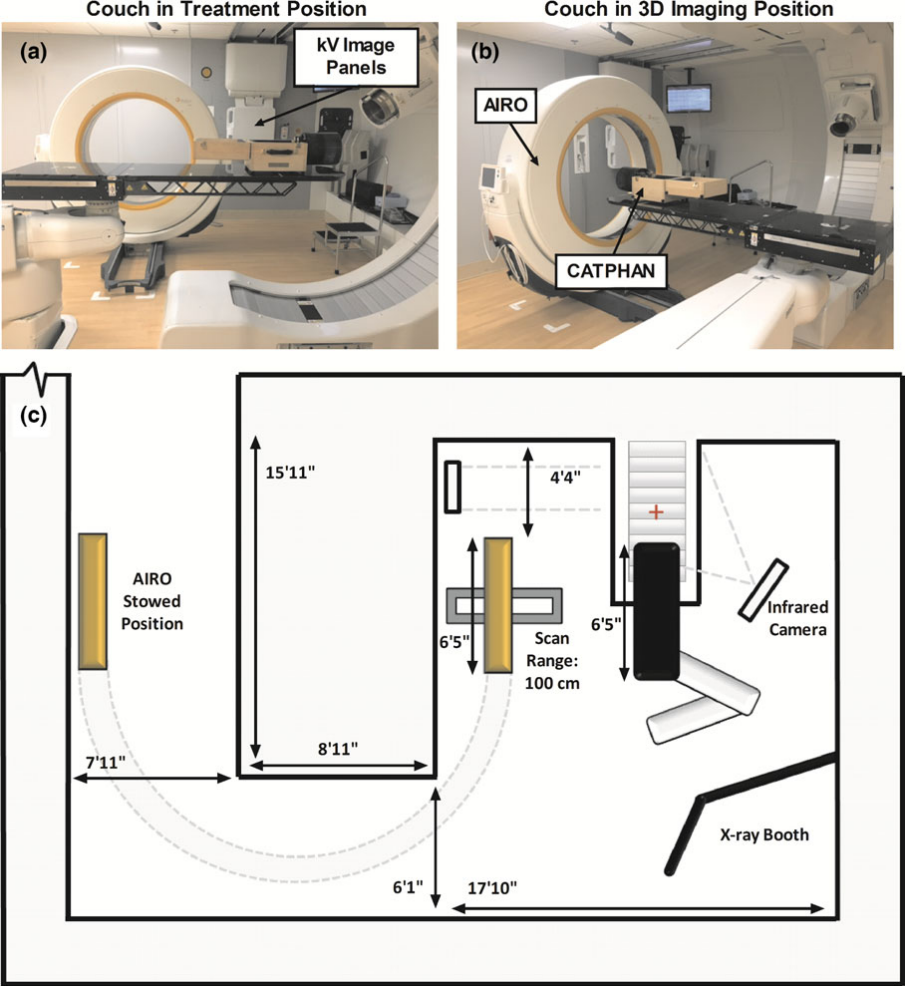
Mevion‐AIRO Room Setup. (a) Treatment room with couch in setup position. (b) Treatment room with couch in imaging position. (c) Birds‐eye‐view of the treatment room. The AIRO is shown in scan mode with 100 cm max scan range and 51.2 cm image field‐of‐view. PA and Left Lateral kV imaging panels are also shown with associated track.

The AIRO Mobile CT System (Mobius Imaging LLC, Shirley, MA, USA) is a large bore (107 cm) helical 32 slice CT scanner historically utilized for intra‐operative imaging for spinal surgeries. Our institution is the first to acquire and clinically implement the AIRO Mobile CT System (AIRO) for IGPT. The AIRO's small footprint (W × L × H: 1.94 × 1.54 × 1.90 m) occupies 1.28 m^2^ of treatment floor space (in scan mode). When not in use, the AIRO is stored in the maze (Fig. 1) to avoid radiation‐induced damage to its sensitive electronics. The AIRO's motor‐controlled castors provide effortless transport from the storage location (in the maze hallway) to the scanning location.

The AIRO's image quality characteristics have been reported by Weir et al. for surgical applications.^13^ Our study assessed the performance characteristics of the AIRO at our institution compared with IGRT systems used for intensity‐modulated radiation therapy (IMRT) and CT simulators at our institution for radiotherapy applications. Our current IGPT workflow involves CT simulation using a Philips Big Bore 16 Slice CT Simulator with routine orthogonal kV/kV image pairs (flat‐panel detectors) preceding each treatment fraction. The AIRO will be utilized for target localization and inter‐fraction adaptive treatment assessment. To our knowledge, our facility is the first compact proton therapy system to use a mobile CT scanner for IGPT.

## 2 Materials and methods

Mevion Medical Systems has developed software, Verity™ 3D, that uses CT images from any scanner to perform 3D image registrations for proton therapy. Due to the ease of motion and small footprint, we have selected the AIRO CT scanner for IGPT. The AIRO is sold commercially for surgical purposes by BrainLab (Munich, Germany). Mevion has developed an infrared camera tracking system to interface with the CT scanner to facilitate use of CT images for IGPT. This system references the 3D image set to the in‐room coordinates and planning CT via a reference frame, infrared camera, and CT scan. This reference frame attaches to the treatment couch and includes infrared markers to determine initial room coordinates as well as ceramic markers to reference the CT image to room coordinates. An additional infrared marker is rigidly attached to the gantry so that changes in the reference frame location in relation to the treatment room can be detected as the robotic couch moves between the initial treatment setup location and the CT imaging location. The AIRO unit was specifically adapted to use inside the proton vault by removing the integrated stand that supports the weight of surgical gurneys in order to avoid interference with the robotic couch (Fig. 1).

### 2.A Image quality characterization and comparison to simulators and IGRT systems

Five CT images of a Catphan^®^504 phantom (The Phantom Laboratory, Salem, NY, USA) were acquired on the AIRO Mobile CT System, a Varian EDGE™ Radiosurgery System (EDGE) On Board Imager, a Philips Brilliance Big Bore 16 slice CT simulator, and a Siemens SOMATOM Definition AS 20 slice CT simulator. Medial and lateral lasers were used to align the phantom prior to image acquisition.

Image acquisition was performed with various protocols. On the AIRO, images were acquired with soft, standard, and sharp reconstruction kernels with the pre‐set clinical head protocol. The AIRO's pre‐set clinical protocols for head, thorax, abdomen, pelvis, shoulder are listed in Table 1. The sharp, standard, and soft kernels are noise filtering Gaussian smoothing kernels with *σ* = 0.00, 0.68, and 0.92, respectively.

**Table 1:** Edge CBCT & AIRO Helical CT clinical pre‐set protocols/techniques

Edge CBCT techniques			AIRO helical CT protocols		
Techniques	kVp	mAs	Protocols	kVp	mAs
Head	100	150	Head	120	326
Thorax	125	268	Thorax	120	68
Pelvis	125	1080	Pelvis	120	149
Pelvis Obese	140	1681	Abdomen	120	149
Image Gently	80	97	Shoulder	120	149

On the EDGE, images were acquired using the following pre‐set clinical techniques: head, thorax, pelvis, pelvis obese, and image gently (Table 1). On the Philips CT simulator, standard clinical protocols were used (120 kVp, 450 mA) with 1.5 mm, 2.0 mm, and 3.0 mm slice thickness. On the Siemens CT simulator images were acquired with standard brain (120 kV, 450 mAs), high res brain (120 kV, 580 mAs), abdomen (120 kV, 250 mAs), and thorax (120 kV, 140 mAs) protocols with 1.5 mm, 2.0 mm, and 3.0 mm slice thickness.

Multimodality comparisons were made using the clinical head protocols on the AIRO, Philips, and Siemens CT simulators and the pelvis technique on the Edge. The pelvis technique was used on the Edge because it uses full 360° gantry rotation and provides the highest image quality. The Edge head protocol involves a 200° gantry rotation and is configured for low dose rather than image quality.

DoseLAB software v.6.6 (Mobius Medical Systems LP, Houston, TX, USA) was used for image quality analysis. Modules CTP404, CTP528, and CTP486 were analyzed. The following image quality measures were assessed: modulation transfer function (MTF), scaling discrepancy, geometric distortion, spatial resolution, overall uniformity, minimum uniformity, contrast, CNR, and maximum HU deviation (Table 2).^14^ The mean and standard deviation of five or more scans are reported. The low contrast module (CTP515) was assessed on the AIRO, Philips, and Siemens scans. Low CNR was calculated using the 15.0 mm 1% Supra‐slice target. A region‐of‐interest (ROI) was dropped onto the Supra‐slice target and another on a uniform region of the phantom.

**Table 2:** Image quality tests and calculation methods

Image quality tests	Definition
Modulation Transfer Function (MTF)^a^	The modulation of multiple ROIs in the phantom with various line bar patterns. As spatial frequency increases, ROI's modulation decreases. The modulation is normalized to its highest value and plotted.
Modulation^a^	$\frac{HU_{90}-HU_{10}}{HU_{90}+HU_{10}}$ Where $HU_{90}$s the 90^th^ percentile CT number in the ROI and $HU_{10}M\left(\%\right)$ s the 10^th^ percentile CT number in the ROI.
Scaling Discrepancy^a^	The maximum possible error in a measurement of distance. $\left|M(\%)-100\%\right|\times L$ Where M(%) is the magnification of the image and L is the length of the image's longest side.
Geometric Distortion^a^	Verifies that correct distance measurement occurs in all regions of the CT image.
Overall Uniformity^a^	$\left(1-\frac{Max_{90}-Min_{10}}{Max_{90}+Min_{10}}\right)\times 100\%$ Where $Max_{90}$ is the maximum 90^th^ percentile CT number in all uniformity regions and $Min_{10}$ is the minimum 10^th^ percentile CT number in all uniformity regions
Minimum Uniformity^a^	The lowest uniformity of all ROIs. Uniformity is calculated as: $\left(1-\frac{HU_{90}-HU_{10}}{HU_{90}+HU_{10}}\right)\times 100\%$ Where $HU_{90}$ is the 90^th^ percentile of the pixel CT number in the ROI and $HU_{10}$ is the 10^th^ percentile of the CT number in the ROI.
Maximum HU Deviation^a^	The maximum deviation between mean HU value of an ROI subtracted from its defined reference HU absolute value of all calculated HU deviation on an image.
Contrast^a^	$\left(\frac{HU_{2}-HU_{1}}{HU_{2}+HU_{1}}\right)\times 100\%$ Where $HU_{2}$ is the mean CT number in the region with greater signal and $HU_{1}$ is the mean CT number in the region with lesser signal.
High Contrast‐to‐Noise Ratio^a^	$\frac{HighContrast}{Noise}=\left(\frac{HU_{2}-HU_{1}}{HU_{2}+HU_{1}}\times 100\%\right)/\left(\frac{\sqrt{\left(\sigma^{2}\right)+{\left(\sigma_{1}\right)}^{2}}}{\sqrt{{\left(HU_{2}\right)}^{2}+{\left(HU_{1}\right)}^{2}}}\right)\times 100\%$ Where $\sigma_{1}$ is the standard deviation of the high contrast ROI, $\sigma_{2}$is the standard deviation of background, $HU_{1}$ is the mean CT number of the high contrast ROI and $HU_{2}$ is the mean CT number of background.
Low Contrast‐to‐Noise Ratio^13^	$\frac{LowContrast}{Noise}=\frac{\left|HU_{ROI}-HU_{b}\right|}{\sigma_{b}}$ Where $\sigma_{b}$ is the standard deviation of the background ROI, $HU_{ROI}$ is the mean CT number inside the low contrast ROI and $HU_{b}$ is the mean CT number of the background ROI.

a Definitions derived from DoseLab v.6.6 User Manual.^17^

### 2.B Localization accuracy

A Stereophan phantom (Sun Nuclear Corporation, Melbourne, FL, USA) was used to measure the localization accuracy of the Mevion‐AIRO system. The universal spacer insert and CT/MRI insert were inserted into the cylinder cavity. The phantom was leveled with the precision leveling stand. Treatment isocenter was placed at the BB target corresponding to the laser alignment marks on the outside of the phantom. The robotic couch was re‐positioned to acquire the AIRO images following the Mevion localization workflow. Once the images were acquired, the robotic couch was moved back to the initial treatment isocenter based on the robotic couch's coordinates. The software then performed a CT registration correlating the reference frame's ceramic fiducials to its infrared markers (relative to treatment isocenter). The images were then manually registered to the 3D planning CT. Successively, a kV/kV image pair was acquired and registered to the digitally reconstructed radiograph (DRR). Localization accuracy was defined as the difference between suggested shifts from the kV/kV pair versus the AIRO CT image set, where the kV/kV image was considered the gold standard. In addition, end‐to‐end tests were performed to illustrate both time and workflow for patient setup and target localization.

Additional localization accuracy tests were performed using the 6 degree‐of‐freedom MIMI Phantom (Standard Imaging, Inc. Middleton, WI, USA). The default Q‐fix overlay couch top was used. With the proton gantry positioned at 55° the reference frame was attached to the couch top and the couch was positioned to 270° to capture the reference frame. Known shifts were applied to the phantom separately including all translational directions (lateral, longitudinal, and vertical), all rotations (yaw, shift, and roll), and cumulatively (all 6 degrees of freedom). A reference kV/kV image set was acquired preceding the AIRO CT image. The couch was moved to the 3D imaging position and a 3D CT image was acquired. The image was sent to the Mevion treatment console and Verity, and then registered to the 3D planning CT. An initial manual rigid registration was performed followed by automated registration for fine tuning. Suggested shifts were applied. The robotic couch was then re‐positioned to standard imaging position and a 2D orthogonal kV x‐ray pair was acquired. Suggested shifts (kV/kV) were recorded. Localization accuracy was defined as the difference between suggested shifts and known shifts.

### 2.C Measured imaging dose

CT Dose Index for helical scans (CTDI_vol_) was calculated using a 16‐cm and 32‐cm cylindrical polymethylmetacrylate phantom (CIRS, Computerized Imaging Reference Systems, Inc., Norfolk, VA, Model 007 & 007A) with 100 mm pencil ionization chamber and electrometer (Raysafe Xi CT Detector, Unfors RaySafe, Inc., Cleveland, OH, USA) on the AIRO and the Philips CT Simulator. The ion chamber was placed at the 3 o'clock and the 12 o'clock positions. Three dose measurements were acquired at each position. Measured dose was then compared to the vendor‐reported CTDI_vol_. ACR requirements specify that dose must be within 20% of manufacturer‐reported specifications.^15^

## 3 Results

### 3.A Image quality characterization and comparison to simulators and IGRT systems

#### 3.A.1 AIRO reconstruction kernel effects on image quality

**Figure 2:**
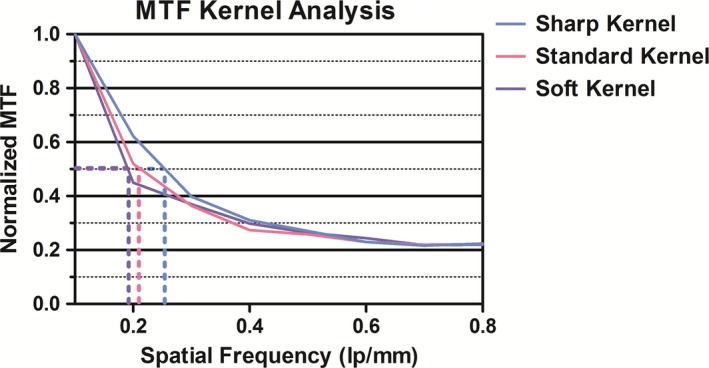
AIRO Normalized MTF for sharp, standard, and soft reconstruction kernels—calculated with DoseLAB v6.6.

The AIRO image quality results for various kernels are listed in Table 3. The AIRO sharp kernel provided the best MTF (0.26 line pairs/mm). High CNR and spatial resolution (31.1% difference) decreased from sharp to soft kernel. Low CNR increased from sharp kernel to soft kernel (5.73–8.51). Overall uniformity and minimum uniformity were comparable across reconstruction kernels (<0.5% difference). Geometric distortion was equal for sharp and standard reconstruction kernels and <7% difference between sharp/soft and standard/soft kernels. Figure 2 illustrates that the MTF for the sharp kernel with small FOV exceeded that of the soft kernel.

**Table 3:** AIRO image quality results for varying reconstruction kernels

	Sharp	Standard	Soft
Scaling discrepancy (mm):	1.19	1.34	1.35
Geometric distortion (mm):	0.14	0.14	0.15
Spatial resolution (50% MTF, lp/mm):	0.26	0.21	0.19
Overall uniformity (%):	98.85	98.94	98.99
Minimum uniformity (%):	99.06	99.19	99.31
Contrast (%):	10.68	10.56	10.41
High CNR:	30.34	21.45	17.49
Low CNR:	5.73	7.12	8.51
Maximum HU deviation (HU):	58.98	57.50	57.68

#### 3.A.1 Comparison to simulators and IGRT systems

**Figure 3:**
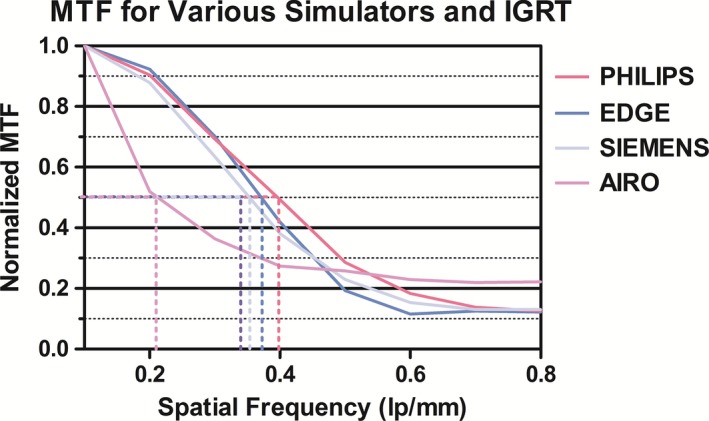
Normalized MTF for Philips CT simulator, AIRO, and EDGE CBCT—calculated with DoseLAB v6.6.

Figure 3 illustrates that the MTF and spatial resolution of the AIRO (0.21 lp/mm) was lower than the Philips CT simulator (0.40 lp/mm), Siemens CT simulator (0.35 lp/mm), and Edge CBCT (0.37 lp/mm). Conversely, the Philips CT simulator exhibited superior MTF and spatial resolution compared to all modalities (Table 4 and Fig. 3). The AIRO had comparable minimum and overall uniformity when compared to the Philips CT simulator, Siemens CT simulator or the Edge (<0.5% difference, Table 4). The Philips high CNR was superior to the AIRO with a 3.65 difference between the two values (18.9% difference). The Siemens high CNR was superior to the AIRO with a 7.02 difference between the two values (28.1% difference). Philips maximum HU deviation was comparable to the AIRO (17.0% difference). Siemens maximum HU deviation was larger than the AIRO's maximum HU deviation (43.5% difference). The Edge demonstrated the smallest HU deviation (21.66 HU, 90.6% difference from AIRO).

**Table 4:** Image quality results—AIRO, Philips CT simulator, Siemens CT simulator and Edge CBCT (DoseLAB)

	Philips	Airo	Siemens	Edge^a^
Scaling discrepancy (mm):	0.21	1.34	0.03	0.86
Geometric distortion (mm):	0.11	0.14	0.11	0.13
Spatial resolution (50% MTF, lp/mm):	0.40	0.21	0.35	0.37
Overall uniformity (%):	99.21	98.94	99.37	98.93
Minimum uniformity (%):	99.25	99.19	99.37	99.26
Contrast (%):	10.30	10.56	12.03	10.79
High CNR:	21.14	21.45	28.47	23.36
Low CNR:	2.00	7.12	2.46	–
Maximum HU deviation (HU):	48.50	57.5	89.49	21.66

a EDGE CBCT images were acquired with the Pelvis protocol.

AIRO/Philips image quality differences were within 3% for overall uniformity, minimum uniformity, and contrast and high CNR. AIRO/Philips geometric distortion difference was 0.03 mm (24%). AIRO/Siemens high CNR differences were 28.1% and geometric distortion differences were 24%. AIRO/Siemens differences exceeded 100% for scaling discrepancy (191.2%). The AIRO low CNR was 7.12 for the standard reconstruction kernel. The Philips and Siemens CT Simulator low CNR were 2.00 and 2.46, respectively. Refer to Table 5 for HU constancy values for the AIRO, Philips CT Simulator, Siemens CT Simulator and Edge CBCT (Module CTP404).

**Table 5:** HU Constancy (CTP404) for various materials in Philips, Edge, Siemens and AIRO

	Philips (HU)	Edge (HU)	Airo (HU)	Siemens (HU)	True (HU)
Acrylic	132.44	123.08	108.15	129.67	100
Air	−969.86	−999.41	−978.49	−1023.34	−1000
Polystyrene	−22.69	−36.62	−47.83	−52.26	−35
LDPE	−79.01	−94.88	−102.08	−119.95	−100
PMP	−167.91	−186.91	−191.44	−223.49	−200
Teflon	941.50	968.34	932.32	1079.30	990
CTP404	103.64	106.06	92.99	96.00	100

### 3.B Localization accuracy of Mevion‐AIRO system

Localization accuracy results using the Stereophan phantom (based on two trials) demonstrated maximum AIRO‐kV/kV shift differences of 0.1 mm in the x‐direction, 0.1 mm in the y‐direction, and 0.2 mm in the z‐direction. (Rotations were not included in the analysis because the target was a single BB inside of the phantom.) The Mimi Phantom was used to characterize the rotational corrections. The RMS values were between 0.22 mm and 0.28 mm. Localization accuracy (based on the MIMI phantom) was within 0.6°. Registration (kV/kV) was within 0.5° and 0.5 mm from 3D/3D registrations. These results included robotic couch motion from the CT scanner imaging position to the standard kV imaging isocenter.

The localization accuracy tests also included a workflow and time analysis to determine the optimal location of the CT scanner in the treatment room and to gauge the time required for a CT scan. The resultant workflow required one therapist to bring the CT scanner into the room while an additional therapist performed the initial patient setup. An additional 5–10 min were needed to acquire a CT scan.

### 3.C CTDI_vol_

Measured CTDI_vol_ for the AIRO, and Philips CT Simulator are illustrated in Table 6. The AIRO had the highest dose especially for the head protocol which gave 27.14 mGy more dose than the Philips CT Simulator. The standard helical AIRO head protocol (120 kV, 240 mA, 326 mAs (effective), 1.92 s rotation time, 1.06 mm slice collimation, 1.0 mm slice width, 1.415 pitch factor, soft kernel) gave 64.7 mGy CTDI_vol_. Measured CTDI_vol_ was 72.05 mGy (Table 6). The helical AIRO abdomen protocol (120 kV, 110 mA, 149 mAs (effective), 1.92 s rotation time, 1.06 mm slice collimation, 1.0 mm slice width, 1.415 pitch factor, standard kernel) gave 22.3 mGy CTDI_vol_. Measured CTDI_vol_ was 26.08 mGy (Table 6). The Philips clinical 1.5 mm head protocol (120 kV, 339 mA, 580 mAs (effective), 0.75 s rotation time, 1.5 mm slice thickness, 0.438 pitch factor) and clinical 3.0 mm body protocol (120 kV, 488 mA, 450 mAs (effective), 0.75 s rotation time, 3.0 mm slice thickness, 0.813 pitch factor) was used. CTDI_w_ was also measured on the EDGE (pelvis protocol, Table 1). Dose measurements were 15.6 mGy for head and 55.3 mGy for abdomen. In comparison, Amer et al. reported CBDI_w_ (cone beam CT dose index) values of 1.6 mGy, 6 mGy and 25 mGy for head, lung, and pelvis CBCT protocols, respectively, on an Elekta Synergy treatment machine.^16^ For the AIRO and the Philips Simulators, dose was within the ACR requirements of 20% of manufacturer‐specifications.

**Table 6:** Measured CTDI_vol_ for AIRO helical CT and Philips CT simulator for head and abdomen protocols

	CTDI_vol_ (mGy)	
	Airo	Philips
HEAD	72.05	44.91
ABDOMEN	26.08	24.09

## 4 Discussion

This study describes the characterization and commissioning of the AIRO mobile CT system for IGPT. Our characterization of the AIRO's image quality demonstrated that the spatial resolution of the AIRO was lower than our other simulators and IGRT systems. Weir et al. reported similar findings about the AIRO indicating its poor spatial resolution when compared to a Siemens Sensation 64 Slice CT scanner. Weir et al. also reported ring artifacts which could not be eliminated. We found similar ring artifacts present when phantoms had widths at or near the width of the detector (32 cm). Ring artifacts were most noticeable at the phantom/air interface in cylindrical phantoms. These artifacts were not present in anthropomorphic phantoms, thus we conclude that ring artifacts should not appear on patient scans. Image quality is protocol and reconstruction kernel dependent. We also found a large scaling discrepancy in comparison to the Edge and simulators. We attribute this difference to the AIRO's large bore. Scaling discrepancy is calculated based on the image's magnification and the length of the longest side of the image.^17^ One last potential disadvantage of the AIRO for daily IGRT is the additional patient dose. We measured AIRO's CTDI_vol_ in the range of 26.1–72.1 mGy. These doses are slightly higher than the values measured for the Edge CBCT (15.6–55.3 mGy), and may need to be taken into account if multiple CT datasets are acquired on a daily basis.

Although the AIRO's image quality is worse than CT simulators and the Edge CBCT, the AIRO's localization accuracy was within 0.6 mm and 0.5° measured using the MIMI phantom. Additionally, we demonstrated that the localization accuracy of the Mevion‐AIRO system using the Stereophan phantom was within 0.2 mm when compared with orthogonal kV/kV pairs. This accuracy is comparable to other commonly used IGPT modalities. Landry et al. reported 1.4 mm localization accuracy for a gantry‐mounted CBCT system^18^ and Xu et al. 2016) reported the accuracy of the Edge localization kV/kV pair to be within ±0.3 mm and ±0.3°.^19^ It is also important to note that the AIRO's localization accuracy included the motion of the robotic couch from treatment isocenter to the CT location. We suggest that CT scanner QA include additional robotic couch tests to insure the AIRO's localization accuracy is not adversely affected by robotic couch movement. Based on this phantom study and initial commissioning, the AIRO can be used for accurate and reliable IGPT.

Although other facilities have acquired mobile helical CT systems, such as the BodyTom (Neurologica Corporation, Danvers, MA, USA) and the Aquilion LB (Toshiba), our proton therapy facility is the first to acquire the AIRO Mobile CT System for proton treatment localization.^9^, ^10^, ^11^ We are also the first to use this technology for patient localization in a small‐compact proton facility where space limitations exist. Based on our study, the AIRO Mobile CT System can be utilized for accurate and safe image‐guided proton therapy. At the time of writing this manuscript, we have commissioned the system and begun clinical use of the system for treatment localization. Our preliminary findings are that an additional 3–5 min of treatment time are required to bring the scanner into the treatment room. Future work will involve a time study for patient localization and evaluation scanning as well as characterization of the CT images as they relate to stopping power and CT number constancy for use in adaptive proton therapy.

## Conclusions

This work evaluated various image quality, localization accuracy, and dosimetric metrics of the AIRO and compared them to other common modalities. Based on our findings, we recommend that the AIRO Mobile CT System can be applied clinically for safe and accurate image‐guided proton therapy.

## conflict of interest

There are no conflicts of interest.
